# P03.04. Attitudes and knowledge of medical students towards complementary therapies

**DOI:** 10.1186/1472-6882-12-S1-P257

**Published:** 2012-06-12

**Authors:** L Shih Min, N Mendes Morales

**Affiliations:** 1 Universidade Federal de Santa Catarina, Florianopolis, SC, Brazil

## Purpose

To evaluate the attitudes and the degree of knowledge of medical students from Universidade Federal de Santa Catarina, Florianopolis, Brazil, towards complementary therapies of the National Policy on Complementary and Integrative Practices of the SUS.

## Methods

Observational transversal quantitative survey using a questionnaire, which was built by the authors and consisted of two parts: the first part contains the student’s personal data, besides some questions about knowledge, self use and willing to recommend the therapies; the second part is about the beliefs and attitudes toward complementary therapies.

## Results

Three hundred ninety-two students (65.2% of all undergraduates) answered the questionnaire, with a uniform distribution between genders. Of these, 30.7% affirmed they had been treated with complementary therapies. Eighty-eight percent knew about complementary therapies with the most known being herbal medicine (90.3%). Eighty- three percent declared they got the knowledge about the therapies from non-academic sources such as media. The majority demonstrated favorable attitudes towards the therapies, and 84.7% of them would recommend or support their use by their patients and family, and 75.3% of them would like to learn about the subject in curricular classes. Few students (15.6%) claimed this subject was taught in undergraduate curricular classes.

## Conclusion

The UFSC medical students demonstrated favorable attitudes towards complementary therapies, willing to recommend and support their use by their patients or family. They were inclined to learn about the subject in curricular classes, especially the students belonging to the central phase (third and fourth years) and among females. The students also revealed they knew about the therapies through non-academic sources and that the teaching level during the medical undergraduate course in UFSC is low.

